# Preoperative frailty assessment could be a predictive factor for the prognosis of elderly patients undergoing coronary artery bypass grafting: a retrospective case-control study

**DOI:** 10.1186/s12871-023-02024-0

**Published:** 2023-02-28

**Authors:** Rui Pan, Xiaohui Li, Jingjing Han, Qian Li, Zheng Lei, He Huang, Yu Chen

**Affiliations:** 1 Department of Anesthesiology, First Affiliated Hospital of Nanjing Medical University, 300 Guangzhou Road, Gulou District, Nanjing City, 210029 Jiangsu Province China; 2 Department of Anesthesiology, the Fourth People’s Hospital of Taizhou, 99 Gulou North Road, Hailing District, Taizhou City,, 225399 Jiangsu Province China

**Keywords:** Preoperative frailty assessment, Coronary artery bypass grafting, Elderly patients, SOFA score, Prognosis

## Abstract

**Background:**

Frailty has been considered to be associated with major mortality and increased length of stay after cardiac surgery. This study aimed to explore the predictive potential of frailty assessment in the prognosis of elderly patients undergoing bypass surgery.

**Methods:**

This study assessed the preoperative frailty according to the Fried’s frailty phenotype, and included 150 frail and 150 non-frail elderly patients (≥ 65 y) who underwent bypass surgery. The present study evaluated the prognosis of elderly patients based on sequential organ failure assessment (SOFA) score, and collected clinical indicators to construct logistic regression models with the prognosis as the dependent variable, to explore the potential predictive ability of preoperative frailty. Moreover, this study focused on the complications and analyzed the relationship between preoperative frailty and postoperative complications.

**Results:**

In the present study, 244 patients were divided into the favorable prognosis group and 56 patients were divided into the unfavorable prognosis group. Logistic regression analysis showed that increased myoglobin and high cardiac function classification were independent risk factors for unfavorable prognosis in elderly patients undergoing bypass surgery. The discrimination of the clinical prediction model was determined by the receiver operating characteristic (ROC) curve, and the area under curve (AUC) was 0.928. After adding preoperative frailty assessment, the AUC was improved to 0.939. This study found a significant correlation between preoperative frailty and postoperative complications, mainly in the circulatory system.

**Conclusion:**

Preoperative frailty assessment could be a predictive factor for the prognosis of elderly patients undergoing coronary artery bypass grafting. According to our study, frailty assessment and appropriate intervention before bypass surgery may be beneficial to the enhanced recovery after cardiac surgery.

**Trial registration:**

The clinical study was approved by the Medical Ethics Committee of The First Affiliated Hospital of Nanjing Medical University (2021-SR-393). All patients signed an informed consent form.

## Introduction

Coronary artery disease (CAD), a major cause of death worldwide, accounted for 45.1% of deaths from heart disease. For several decades, coronary artery bypass grafting (CABG) is the standard procedure for revascularization of severe left main or three-vessel coronary artery disease [1]. An increasing number of elderly patients are now undergoing coronary artery bypass surgery [2]. Though the outcomes of CABG surgery have been improved compared to the past [3], elderly patients show a decline in functioning across multiple physiological systems, accompanied with a greater burden of risk factors, which is associated with an increase in complications and prolonged length of stay [4, 5]. Therefore, the prognosis evaluation of CABG has been paid more attention by clinicians.

At present, accurate diagnostic criteria and consensus definitions for clinical prognosis of patients in the intensive care unit (ICU) mainly include acute physiology and chronic health evaluation (APACHE-II), sequential organ failure assessment (SOFA) score and systemic inflammatory response syndrome (SIRS) criteria. Among them, the SOFA score developed by the European Society of Intensive Care Medicine (ESICM) was widely used to reflect worsening organ dysfunction and was proposed as an alternative to other assessments of multiple organ dysfunction [6–8]. Previous study showed that SOFA score had greater prognostic accuracy for in-hospital mortality and was widely used as an effectively predictive tool among patients in the intensive care unit (ICU) [9]. In the acute heart failure patients, the SOFA score could predict long-term mortality [10]. However, the current prognostic scoring system for patients in ICU mainly focuses on the occurrence of postoperative complications, which has limited effect on predicting the occurrence of complications in advance. Therefore, it is fundamental to find a predictive method that can improve the prognosis of patients undergoing CABG surgery.

Frailty is generally defined as a biological syndrome of decreased reserve and resistance to stressors, which resulted from cumulative declines of multiple physiologic systems and caused vulnerability to adverse outcomes [11]. Originally, frailty assessment was used to evaluate the physiological status and survival status of the elderly in the community. The prevalence of frailty has been estimated to be 10–60% in patients with cardiovascular disease [12]. As the population aged, the number of elderly patients undergoing surgery is increasing in recent years. Frailty assessment is gradually gaining popularity as a tool to perform preoperative status assessment. At present, frailty has been considered to be associated with major mortality and increased length of stay after cardiac surgery [13, 14]. Although various measurement tools for preoperative frailty were developed and showed promising ability to predict morbidity and mortality, the Fried’s frailty phenotype has been widely used and validated in both community-dwelling and hospitalized patients [15, 16]. Previous studies had found that the critically ill patients with poor ICU outcomes (SOFA scores higher than 5.65) were associated with frailty [17].

Therefore, this study evaluated the prognosis of CABG patients based on SOFA score scale, and assessed the preoperative frailty status of patients according to the Fried’s frailty phenotype, so as to explore the potential relationship between preoperative frailty and postoperative prognosis. The present study aimed to determine whether preoperative frailty assessment could be a predictive tool for postoperative severe mortality and complications of patients undergoing CABG.

## Methods

### Study participants

The clinical experiment was designed according to the principles of the prospective specimen collection and retrospective blinded evaluation (PRoBE) design [18]. The protocols for clinical experiment were approved by the Medical Ethics Committee of The First Affiliated Hospital of Nanjing Medical University (2021-SR-393). The results of the clinical experiment adhere to the applicable Strengthening the Reporting of Observational Studies in Epidemiology (STROBE) guidelines [19].

Sample size calculation: according to the principle of sample size calculation in logistic regression analysis, the sample size should be 5–10 times of the possible influencing factors. In this study, there were 20 factors affecting the prognosis of CABG patients according to the results of relevant literature, and the sample size was at least 200 cases when calculated by 10 times [20].

**Fig. 1:**
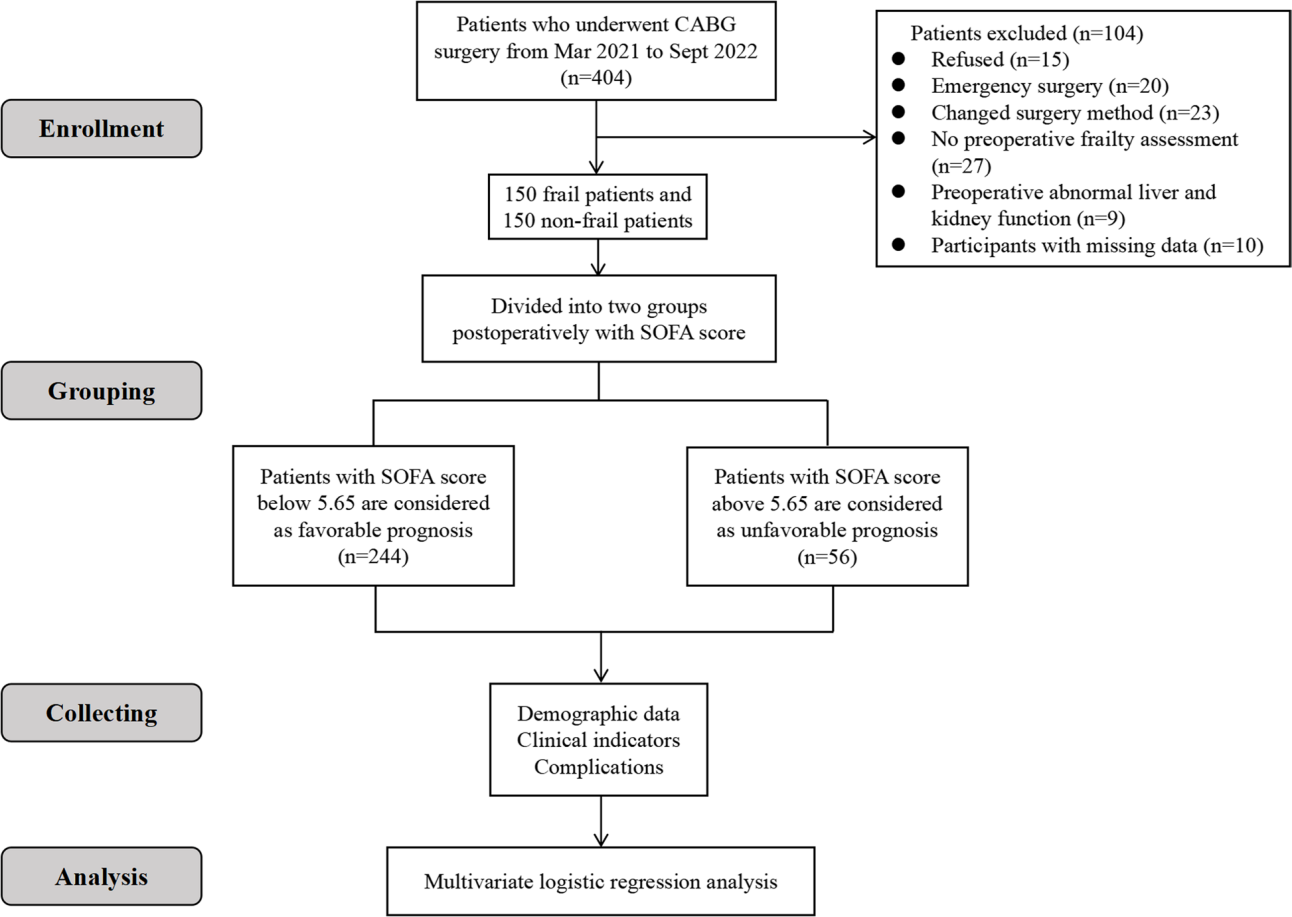
Flow chart of clinical experimentation

As is shown in Fig. 1, a total of 404 patients who underwent CABG surgery at the First Affiliated Hospital of Nanjing Medical University from March 2021 to September 2022 were enrolled in this study. The inclusion criteria were as follows: (1) ≥65 years old; (2) patients underwent CABG surgery; (3) American Society of Anesthesiologists (ASA) physical status of II-III and (4) all patients provided authorization for the use of their medical records for research purposes. The following were the exclusion criteria: (1) refused to sign the informed consent; (2) emergency CABG surgery; (3) changed surgery method; (4) no preoperative frailty assessment; (5) preoperative abnormal liver and kidney function and (6) participants with missing data. Finally, 150 frail and 150 non-frail patients were enrolled in this study.

### Perioperative management

In the operating room, general anesthesia was induced with fentanyl, midazolam, propofol and was maintained with sevoflurane and cisatracurium. CABG was performed with or without cardiopulmonary bypass (on-pump or off-pump surgery) according to the discretion of the operator. All the operations were performed by one experienced surgical team. The grafts were internal mammary artery and saphenous vein. The coronary arteries involved in the bypass surgery were mainly left anterior descending, diagonal branch and left circumflex artery. Hemodynamics were maintained with vasoactive drugs and antiarrhythmic drugs, and the mean arterial pressure was maintained at 60–80 mmHg. The body temperature was controlled at 36–37 °C without cardiopulmonary bypass (CPB), and was controlled at 30–32 °C during CPB. In the ICU, patients were routinely monitored and generally sedated with propofol or midazolam. Mean arterial pressure targets in the ICU were 60–80 mmHg depending on the procedure and surgeon.

### Outcome assessment and grouping principle

The SOFA score was used to evaluate the related clinical indicators and complications of 300 patients after operation, including the circulatory, respiratory, blood, nervous, liver and kidney system. According to the previous literature, the score of more than 5.65 was considered as unfavorable prognosis group and the score of less than 5.65 was considered as favorable prognosis group [17].

### Preoperative frailty assessment

Baseline frailty assessments were performed using the validated Fried’s frailty phenotype evaluating 5 domains [15]: (1) Shrinking, defined as unintentional weight loss of ≥10 pounds in the last year; (2) Weakness, determined by grip strength, adjusted for gender and body mass index (BMI); (3) Exhaustion, determined by 2 questions from the modified 10-item Center for Epidemiological Studies-Depression scale [21]; (4) Low physical activity, determined by the modified Minnesota Leisure Time Activities Questionnaire [22]; (5) Slowed walking speed, as measured at normal pace over 15 ft. Each domain can obtain a score of 0 or 1 based on previous description. In the present study, patients with a total score of 3–5 were defined as frailty before surgery.

### Data collection

All researchers received systematic training before the start of this study. The 2 researchers responsible for the primary interview were certified in National Drug Clinical Trial Institute. All the data of the patients undergoing CABG were collected from the hospital’s electronic medical records. (1) General information were recorded, including gender, age, frailty assessment, hypertension classification, cardiac function classification, ASA classification, diabetes mellitus and prior stroke. (2) Preoperative data included: the proposed operation method (on-pump or off-pump), myocardial markers, blood routine indicators, biochemical indicators and ejection fraction (EF). (3) Intraoperative data included: surgical duration and anesthesia duration. (4) Postoperative indicators included: postoperative extubation time, length of ICU stay, length of postoperative hospital stay and mortality.

### Statistical analysis

The differences between two groups were analyzed using t tests for normally distributed variables; otherwise, the Mann-Whitney U test was used. Differences in categorical variables were tested using the χ2 test or Fisher exact test. *P < 0.05 and **P < 0.01 were considered statistically significant. Logistic regression was used to examine the relationships among preoperative data, preoperative frailty assessment and prognosis. Preoperative data were entered as independent variables in a binary logistic forward stepwise regression analysis to assess associations with the outcome variable. Discrimination of the prediction models with and without frailty assessment were assessed using the area under the receiver operating characteristic curve (AUC). Statistical analyses were performed using IBM SPSS Statistics (version 25.0).

## Results

### Participants grouping

In this study, the prognosis of patients was evaluated based on SOFA score, 244 patients were divided into the favorable prognosis group and 56 patients were divided into the unfavorable prognosis group. Compared with the unfavorable prognosis group, the postoperative extubation time (1.00 [1.00, 3.00] vs 9.00 [5.00, 23.75], *P* = 0.000), length of ICU stay (4.00 [3.00, 6.00] vs 14.50 [8.00, 27.50], *P* = 0.000) and length of postoperative hospital stay (10.00 [7.00, 13.00] vs 19.50 [11.25, 37.75], *P* = 0.000) in the favorable prognosis group were significantly shortened. The top three postoperative complications in the unfavorable prognosis group are hypotension, anemia and postoperative cognitive dysfunction. Compared with the unfavorable prognosis group, the incidence of hypotension (118, 48.40% vs 53, 94.60%, *P* = 0.000), anemia (81, 33.20% vs 52, 92.90%, *P* = 0.000) and postoperative cognitive dysfunction (42, 17.20% vs 50, 89.30%, *P* = 0.000) in the favorable prognosis group was significantly reduced.

### Clinical characteristics and demographics of participants

There is no significant difference in most of preoperative variables between the patients with favorable and unfavorable prognosis. In the hypertension classification, cardiac function classification and ASA classification, these results were more severe in the unfavorable prognosis group (*P* < 0.05) (Table 1).

**Table 1:** Clinical characteristics and demographics of the patients

	Favorable prognosis (*n* = 244)	Unfavorable prognosis (*n* = 56)	*P* value
Age, y	71 (68, 75)	71 (69, 76)	0.371
Gender, n (%)			0.443
Male	175 (71.72)	43 (76.79)	
Female	69 (28.28)	13 (23.21)	
Diabetes mellitus, n (%)	93 (38.11)	27 (48.21)	0.164
Prior stroke, n (%)	70 (28.69)	21 (37.50)	0.196
Hypertension classification, n (%)			0.000**
No hypertension	65 (26.64)	11 (19.64)	
I	20 (8.20)	0 (0.00)	
II	98 (40.16)	15 (26.79)	
III	61 (25.00)	30 (53.57)	
Cardiac function classification, n (%)			0.000**
I	30 (12.30)	0 (0.00)	
II	138 (56.55)	10 (17.86)	
III	68 (27.87)	28 (50.00)	
IV	8 (3.28)	18 (32.14)	
ASA classification, n (%)			0.002**
II	97 (39.76)	10 (17.86)	
III	147 (60.24)	46 (82.14)	
Operation method, n (%)			0.006**
Off-pump	177 (72.54)	30 (53.57)	
On-pump	67 (27.45)	26 (46.43)	

Data are presented as number (%) or medians (interquartile range)

**P < 0.01

After comparison of preoperative clinical indicators, TnT, MyO, CK-MB, Hb, Alb, Cr and EF showed significant differences between the two groups. The preoperative values of TnT, MyO, CK-MB and Cr in the unfavorable prognosis group were increased, while the values of Hb, Alb and EF were decreased (Table 2).

**Table 2:** Preoperative clinical indicators of the patients

	Favorable prognosis (*n* = 244)	Unfavorable prognosis (*n* = 56)	*P* value
TnT, ng/L	23.61 (13.80, 79.09)	45.07 (18.72, 159.53)	0.003**
TnI, n (%)			0.549
Negative	150 (61.48)	32 (57.14)	
Positive	94 (38.52)	24 (42.86)	
MyO, ng/mL	34.88 (25.00, 46.50)	69.83 (59.52, 81.47)	0.000**
CK-MB, ng/mL	1.99 (1.25, 3.36)	2.71 (1.67, 4.57)	0.000**
Hb, g/L	130.04 ± 15.77	120.18 ± 20.72	0.001**
Plt, 10^9/L	194.00 (159.00, 233.00)	174.00 (158.00, 219.25)	0.072
Alb, g/L	38.30 ± 3.23	36.03 ± 3.94	0.000**
Tbil, umol/L	11.40 (8.95, 15.28)	12.20 (9.08, 16.28)	0.462
Cr, umol/L	74.65 (64.15, 89.80)	84.85 (75.43, 112.90)	0.000**
Urea, mmol/L	6.26 (5.24, 7.64)	6.99 (5.52, 8.83)	0.059
Lac, mmol/L	0.80 (0.63, 1.18)	1.00 (0.70, 1.38)	0.059
EF, %	61.90 (58.57, 63.70)	58.57 (54.03, 62.70)	0.008**

Data are presented as number (%), means ± standard deviations, or medians (interquartile range)

Abbreviations: *TnT* troponin T, *TnI* troponin I, *MyO* myoglobin, *CK-MB* creatine Kinase Isoenzyme-MB, *Hb* hemoglobin, *Plt* platelet, *Alb* albumin, *Tbil* total bilirubin, *Cr* creatinine, *Lac* lactic acid, *EF* ejection fraction

**P < 0.01

### Preoperative frailty assessment

The Fried’s frailty phenotype was further used to evaluate the incidence of preoperative frailty in elderly patients undergoing CABG surgery. The results showed that in the unfavorable prognosis group, the incidence of preoperative frailty was 92.86% (52/56), while in the favorable prognosis, the incidence of preoperative frailty was only 40.16% (98/244) (*P* = 0.000).

### Discrimination of clinical models

**Fig. 2:**
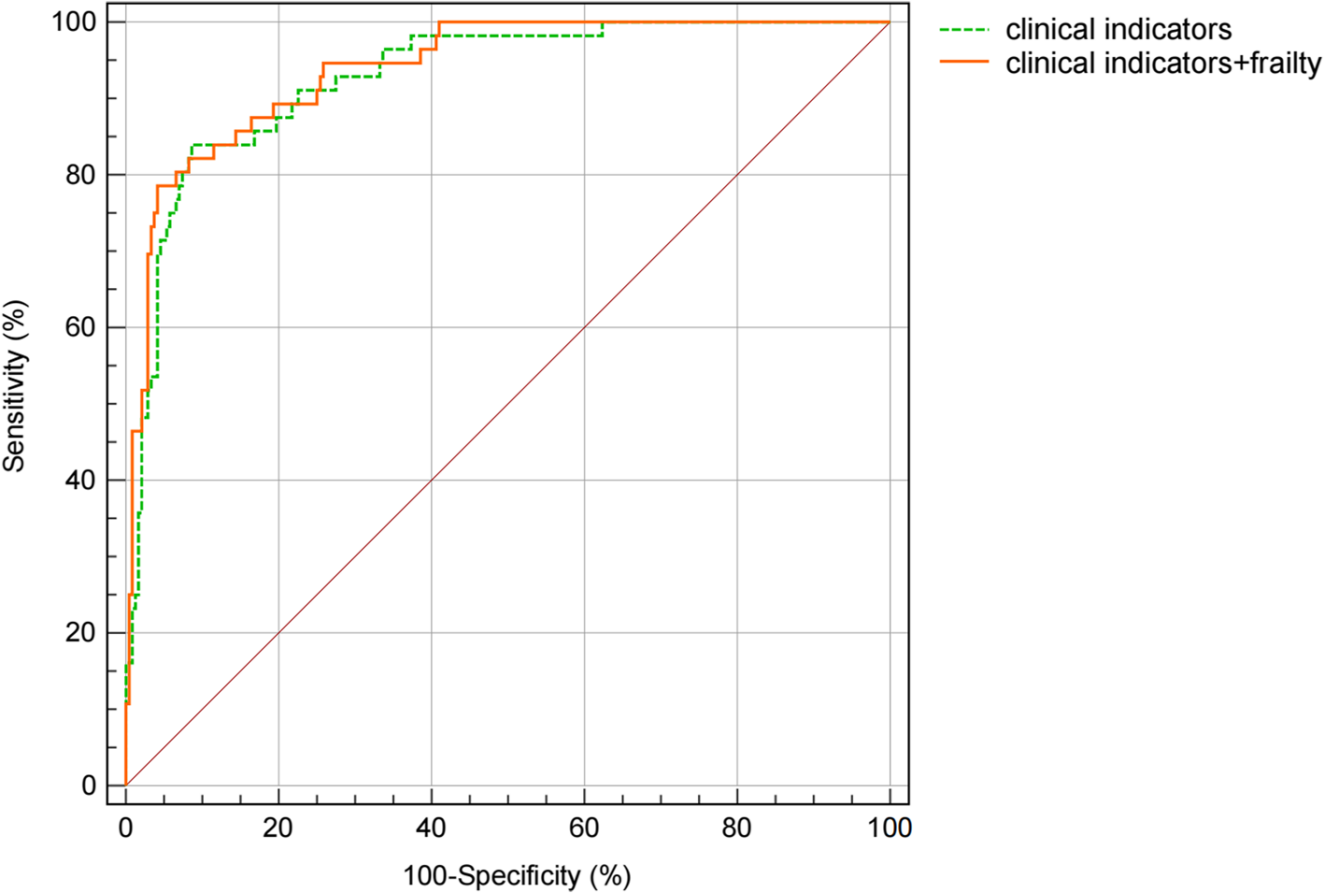
ROC curves of the clinical prediction models. Logistic regression classifier including myoglobin and cardiac function classification: AUC = 0.928 (95% CI, 0.893–0.963). Logistic regression classifier as above, but including frailty: AUC = 0.939 (95% CI, 0.909–0.970). AUC indicates area under the receiver operating characteristic curve; CI, confidence interval; ROC, receiver operating characteristic

Next, we constructed logistic regression model with the prognosis as the dependent variable and 11 statistically different preoperative indicators in Table 1 and Table 2 as covariates. The 11 statistically different preoperative indicators included hypertension classification, cardiac function classification, ASA classification, operation method, TnT, MyO, CK-MB, Hb, Alb, Cr and EF. The myoglobin and cardiac function classification were regarded as predictors of the prognosis in patients under CABG surgery (Table 3). The discrimination of the model was expressed as the receiver operating characteristic (ROC) curve, and the area under curve (AUC) of basic prediction model was 0.928 in the present study (Fig. 2). After adding preoperative frailty assessment on the basis of the basic prediction model (Table 3), the discrimination of the new predictive model was improved by 0.012 (AUC = 0.939) (Fig. 2). These results suggested that preoperative frailty assessment could improve the predictive ability in prognosis of elderly patients undergoing CABG surgery.

**Table 3:** Prediction models for prognosis in elderly patients with coronary artery bypass grafting

Model	Logistic regression analysis			
	B	OR (95%CI)	Wald	***P*** value
Clinical model without frailty (AUC = 0.928 [0.893–0.963])				
MyO, ug/L	0.012	1.084 (1.058, 1.111)	42.772	0.000
Cardiac function classification, n (%)	0.299	4.103 (2.283, 7.373)	22.289	0.000
Clinical model with frailty (AUC = 0.939 [0.909–0.970])				
MyO, ug/L	0.084	1.088 (1.060, 1.116)	40.096	0.000
Frailty, n (%)	2.253	9.519 (2.393, 37.869)	10.229	0.001
Cardiac function classification, n (%)	0.957	2.603 (1.364, 4.966)	8.419	0.004

AUC shown as area ± standard error (95% CI)

Abbreviations: *AUC* area under the receiver operating characteristic curve, *CI* confidence interval

### Meaningful clinical indicators related to frailty assessment

In order to observe the relationships between these meaningful clinical indicators further, we grouped again with preoperative frailty assessment. In addition, we analyzed the correlation between frailty assessment and 11 statistically different preoperative indicators in Table 1 and Table 2, it was found that preoperative frailty was associated with TnT, MyO, CK-MB, Hb, Alb, Cr, EF, hypertension classification, cardiac function classification, ASA classification, and preoperatively proposed operation method (Table 4). It is obvious that statistically different clinical indicators are aggregated in the circulatory system. The indicators such as TnT, MyO, CK-MB, hypertension classification, and EF may be representative indicators of cardiac function in frail patients.

**Table 4:** Meaningful clinical indicators related to frailty assessment

	Non-frail (*n* = 150)	Frail (*n* = 150)	*P* value
TNT, ng/L	21.11 (11.69, 98.51)	31.05 (15.11, 93.22)	0.008**
MyO, ng/mL	31.83 (24.87, 42.28)	49.21 (34.11, 67.63)	0.000**
CK-MB, ng/mL	1.80 (1.18, 3.17)	2.30 (1.39, 3.66)	0.021*
Hb, g/L	130.94 ± 15.48	125.46 ± 18.42	0.006**
Alb, g/L	38.29 ± 3.09	37.47 ± 3.80	0.040*
Cr, umol/L	73.30 (63.60, 85.15)	81.05 (69.30, 102.38)	0.000**
EF, %	62.10 (58.90, 64.00)	60.05 (56.98, 63.00)	0.001**
Hypertension classification, n (%)			0.000**
No hypertension	47 (31.33)	29 (19.33)	
I	17 (11.33)	3 (2.00)	
II	65 (43.34)	48 (32.00)	
III	21 (14.00)	70 (46.67)	
Cardiac function classification, n (%)			0.000**
I	30 (20.00)	0 (0.00)	
II	103 (68.67)	45 (30.00)	
III	16 (10.67)	80 (53.33)	
IV	1 (0.66)	25 (16.67)	
ASA classification, n (%)			0.040*
II	62 (41.33)	45 (30.00)	
III	88 (58.67)	105 (70.00)	
Operation method, n (%)			0.000**
Off-pump	119 (79.33)	88 (58.67)	
On-pump	31 (20.67)	62 (41.33)	

Data are presented as number (%), means ± standard deviations, or medians (interquartile range)

*P < 0.05. **P < 0.01

### Preoperative frailty assessment and postoperative complications

Currently, the relationship between preoperative frailty and postoperative complications remains unclear. This study found there were statistically significant differences in the incidence of all the postoperative complications between the two groups (Table 5). These findings indicated that preoperative frailty was manifested as the decrease of the function in each system, and could be regarded as a concomitant indicator of the decline of the function of each system. Further study found that among 150 frail patients combined with coronary heart disease, up to 108 patients developed hypotension, 51 patients developed arhythmia including atrial and ventricular arrhythmia, and 26 patients died of sudden cardiac arrest (Table 5). The main reason may be elderly frail patients with CAD were often accompanied with symptoms of cardiac dysfunction, so the body was unable to meet the physical needs after stimulation of CABG surgery. In conclusion, the above results further demonstrated that preoperative frailty assessment could be used as an effective method to predict postoperative outcome in elderly patients undergoing CABG surgery.

**Table 5:** Preoperative frailty assessment and postoperative complications

		Non-frail (*n* = 150)	Frail (*n* = 150)	*P* value
Circulatory system	Hypotension, n (%)	63 (42.00)	108 (72.00)	0.000**
	Arrhythmia, n (%)	9 (6.00)	51 (34.00)	0.000**
	Sudden cardiac arrest, n (%)	1 (0.67)	26 (17.33)	0.000**
Blood system	Anemia, n (%)	41 (27.33)	92 (61.33)	0.000**
	Low platelet count, n (%)	37 (24.67)	95 (63.33)	0.000**
Respiratory system	Acute respiratory distress syndrome, n (%)	20 (13.33)	67 (44.67)	0.000**
Nervous system	Postoperative cognitive dysfunction, n (%)	23 (15.33)	69 (46.00)	0.000**
Immune system	Infection, n (%)	17 (11.33)	79 (52.67)	0.000**
Liver system	Hyperbilirubinemia, n (%)	22 (14.67)	42 (28.00)	0.005**
Kidney system	High creatinine, n (%)	21 (14.00)	55 (36.67)	0.000**

Data are presented as number (%). Hypotension is defined as mean arterial pressure lower than 70 mmHg. Arrhythmia refers to the occurrence of postoperative atrial and ventricular arrhythmia. Sudden cardiac arrest refers to the sudden cessation of heart beating after surgery, regardless of whether it can be rescued after cardiopulmonary resuscitation. Anemia is a condition in which a hemoglobin level is less than 70 g/L and blood transfusion is required. Low platelet count is defined as a platelet count of less than 100*10^9/L. Acute respiratory distress syndrome is defined by an oxygenation index (PaO2/FiO2) of less than 300. Postoperative cognitive dysfunction refers to the occurrence of delirium and unconsciousness after surgery. Infection is defined as an increased white-cell count and a positive bacterial culture. Hyperbilirubinemia is defined as a bilirubin value higher than 34.2 umol/L. High creatinine is defined as a creatinine value higher than 133 umol/L

**P < 0.01

## Discussion

Previous study showed that SOFA score had great prognostic accuracy for in-hospital mortality and was widely used as an effective tool among patients in the intensive care unit (ICU) [9]. In this study, among 244 patients in the favorable prognosis group, 1 patient died after CABG surgery. There were 56 patients in unfavorable prognosis group, and 26 patients died after CABG surgery. These results indicated that SOFA score was accurate in grouping the prognosis of CABG patients, which was consistent with the conclusion of previous literature [17]. However, the current clinical prognosis scoring system mainly focuses on the occurrence of postoperative complications, so it is particularly important to seek a predictive evaluation method.

Frailty describes the biological syndrome of decreased physiologic resilience and reserve, it causes a heightened vulnerability to adverse outcomes after an acute stressor. Studies have shown that frailty is associated with increased mortality, surgical site infections, length of hospital stay, increased healthcare expenditure, and readmission rates [23]. Elderly patients, are often complicated with decreased functions of circulatory, respiratory, nervous and other systems, leading to the high incidence of preoperative frailty and more postoperative complications. The pathological manifestations of the circulatory system in frail CAD patients are that insufficient coronary blood supply affects myocardial nutrition, leading to reduced cardiac ejection function. When physiological reserve is insufficient, postoperative cardiovascular complications will increase, such as hypotension, arrhythmia, sudden cardiac arrest and so on (Table 5). In respiratory system, the respiratory muscle strength and alveolar surface area have decreased in frail patients, and pulmonary ventilation and oxygenation function impaired, acute respiratory distress syndrome is more common in frail patients after major surgical trauma. The decrease in the number of neurons in the aged brain is another important clinical features of frailty [24]. Previous studies have shown that the incidence of postoperative cognitive dysfunction is significantly increased in elderly patients [25]. In addition, elderly patients with frailty are often complicated with liver and kidney insufficiency, infection, anemia and other mixed conditions, which form a vicious cycle and lead to unfavorable prognosis [26, 27].

According to our findings in the study, the incidence of preoperative frailty was 92.86% in the unfavorable prognosis group, while the incidence of preoperative frailty was 40.16% in the favorable prognosis. It is suggested that preoperative frailty assessment is necessary in elderly patients undergoing CABG surgery. Logistic regression analysis showed that increased myoglobin and high cardiac function classification were independent risk factors for unfavorable prognosis in elderly patients undergoing CABG surgery. The AUC of the prediction model was 0.928 and was improved by 0.012 to 0.939 after adding preoperative frailty assessment. Therefore, these results suggested that preoperative frailty assessment in elderly CABG patients is meaningful to improve the predictive ability of postoperative outcome.

This study found that increased myoglobin was one of the independent risk factors for unfavorable prognosis in elderly patients undergoing CABG surgery. Myoglobin, an oxygen-binding protein that contains heme, which binds and releases oxygen molecules, and is found mainly in the cells of heart muscle and skeletal muscle. Due to the small molecular weight, myoglobin can be quickly released from damaged cells [28]. According to the 2021 ESC guidelines for the diagnosis and treatment of acute and chronic heart failure, loss of skeletal muscle mass and strength is a late manifestation of heart failure. In the process of skeletal muscle loss, a large amount of myoglobin is released, especially when the cardiac function classification is III, IV. It is suggested that myoglobin is increased to different degrees in each stage of heart failure. Myoglobin can reflect the severity of heart failure and predict the prognosis of patients [29]. The clinical model in this study showed that high myoglobin was an independent risk factor for unfavorable prognosis in patients after CABG surgery. In addition, frailty status was closely associated with high myoglobin in elderly CAD patients (Table 4), which further suggested that preoperative frailty assessment could be a predictive factor for the prognosis of elderly patients undergoing CABG surgery.

After analyzing the intraoperative data of the favorable prognosis group and unfavorable prognosis group, it was found that the surgical duration and anesthesia duration in the favorable prognosis group were 260 min and 325 min, while the surgical duration and anesthesia duration were 280 min and 350 min in the unfavorable prognosis group respectively. The main reason may be patients combined with frailty and cardiac dysfunction mainly underwent on-pump CABG surgery, which led to longer surgical duration and anesthesia duration. Cardiopulmonary bypass procedure can also be considered as an important factor to increase postoperative complications.

This study also has some limitations. First of all, this study was a retrospective study and lacked external validation. In the follow-up study, we plan to conduct a prospective study to confirm the predictive ability of frailty for prognosis. In addition, this study mainly focused on the relationship between preoperative frailty assessment and prognosis during hospitalization, and the follow-up time was short. Furthermore, this study focused only on patients undergoing CABG surgery, which may lead to limitations in the ability of frailty assessments to predict outcomes in patients undergoing other cardiac surgeries.

## Conclusion

Preoperative frailty assessment could be a predictive factor for the prognosis of elderly patients undergoing coronary artery bypass grafting. According to our study, frailty assessment and appropriate intervention before bypass surgery may be beneficial to the enhanced recovery after cardiac surgery.

## Data Availability

The datasets generated and/or analyzed during the current study are not publicly available due to policy issues in the hospital but are available from the corresponding author on reasonable request.

## Abbreviations

CAD: Coronary artery disease
CABG: Coronary artery bypass grafting
ICU: Intensive care unit
APACHE-II: Acute physiology and chronic health evaluation
SOFA: Sequential organ failure assessment
SIRS: Systemic inflammatory response syndrome
ESICM: European Society of Intensive Care Medicine
ASA: American Society of Anesthesiologists
CPB: Cardiopulmonary bypass
ROC: Receiver operating characteristic
AUC: Area under curve
TnT: Troponin T
TnI: Troponin I
MyO: Myoglobin
CK-MB: Creatine kinase isoenzyme-MB
Hb: Hemoglobin
Plt: Platelet
Alb: Albumin
Tbil: Total bilirubin
Cr: Creatinine
Lac: Lactic acid
EF: Ejection fraction
CI: Confidence interval
